
JOURNAL INFORMATION
==============================
NLM Title Abbreviation: Natl Sci Rev
Journal ID: Natl Sci Rev
NLM Journal ID: 101633095
Journal ID: nsr

National Science Review

ISSN: 2095-5138
EISSN: 2053-714X

ARTICLE INFORMATION
==============================
PMCID: PMC7543445
PMID: 34676082
DOI: 10.1093/nsr/nwaa243
Article ID: nwaa243
Article version: 1
Subjects: Perspectives, AcademicSubjects/MED00010, AcademicSubjects/SCI00010

Comparing containment measures among nations by Epidemiological Effects of COVID-19

Gu Jia Center for Statistical Science, Peking University, China

Yan Han School of Mathematical Sciences, Sichua University, China

Huang Yaxuan Yuanpei College, Peking University, China

Zhu Yuru Center for Statistical Science, Peking University, China

Sun Haoxuan Center for Data Science, Peking University, China

Qiu Yumou yumouqiu@iastate.edu Department of Statistics, Iowa State University, USA

http://orcid.org/0000-0002-2338-0873 Chen Song Xi csx@gsm.pku.edu.cn Center for Statistical Science, Peking University, China
Guanghua School of Management, Peking University, China

Corresponding author. E-mail: csx@gsm.pku.edu.cnCorresponding author. E-mail: yumouqiu@iastate.edu
Electronic publication date: 2020 Sep 19
Electronic Location ID: nwaa243
Copyright: © The Author(s) 2020. Published by Oxford University Press on behalf of China Science Publishing & Media Ltd.
Copyright year: 2020
License: This is an Open Access article distributed under the terms of the Creative Commons Attribution License (http://creativecommons.org/licenses/by/4.0/), which permits unrestricted reuse, distribution, and reproduction in any medium, provided the original work is properly cited.
License URL: https://creativecommons.org/licenses/by/4.0/

edited-state: accepted-manuscript
article-lifecycle: PAP

==============================
PERSPECTIVE

### Comparing Containment Measures among Nations by Epidemiological Effects of COVID-19

Jia Gu,[1] Han Yan,[2] Yaxuan Huang,[3] Yuru Zhu,[1] Haoxuan Sun,[4] Yumou Qiu,[5] Song Xi Chen[61]

[1]Center for Statistical Science, Peking University, China
[2]School of Mathematical Sciences, Sichua University, China
[23]Yuanpei College, Peking University, China
[4]Center for Data Science, Peking University, China
[5]Department of Statistics, Iowa State University, USA
[6]Guanghua School of Management, Peking University, China

To whom correspondence should be addressed S. X. Chen at csx@gsm.pku.edu.cn and Y. Qiu at yumouqiu@iastate.edu

The Corona Virus Disease 2019 (COVID-19) has evolved to a global pandemic since its inception in December2019. Countries have responded to the epidemics with different levels of responses and containment measures. Given the unprecedented pressure on nations' healthcare systems and the deaths so far, there is an urgent need to evaluate the effects of the containment measures, which would be useful for countries to plan for their responses to counter the first or possibly the second wave of the epidemic. There have been studies for the effects of COVID-19 control measures taken in China on disease transmission and public health interventions for Wuhan's outbreaks (1–4), and Wuhan travel ban on the spread of COVID-19 both inside China and internationally (5, 6).

There are cross-country studies on COVID-19 pandemics. The effective reproduction numbers between the early-phase and late-phase of COVID-19 outbreaks in 25 international locations (China mainland was not included) were compared with New Zealand's four tier system in (7). This approach is based on estimating time-varying growth rate of confirmed cases rather than a dynamic epidemiological model. Estimation of the effective start dates for non-pharmaceutical interventions (NPI) of 11 European counties and Wuhan, China was considered in (8), which concluded that the effective start of the NPIs occurred five or more days after the official start date of intervention. The effective reproductive numbers of seven Latin American countries were compared with those of Spain and Italy in (9). This study focused on the first 10 days of the local transmission. The effects of travel restriction on COVID-19 in 27 European countries were analyzed based on a constant coefficient SEIR model and a mobility model in (10).

ORIGINAL UNEDITED MANUSCRIPT

© The Author(s) 2020. Published by Oxford University Press on behalf of China Science Publishing & Media Ltd. This is an Open Access article distributed under the terms of the Creative Commons Attribution License (http://creativecommons.org/licenses/by/4.0/), which permits unrestricted reuse, distribution, and reproduction in any medium, provided the original work is properly cited.

Our study is focused on 25 countries which had experienced the COVID-19 epidemic earlier in the pandemic such that they have experienced at least four weeks of established community infections by April 20th, 2020. It is conducted by evaluating and comparing the effective reproduction number $R_t$ curves of the countries and associating them with the timing and the extend of the control measures taken by those countries. The study is based on an extended SEIR model (11) with time varying coefficients (vSEIdR model). Unlike the conventional SEIR model (12), the vSEIdR model allows (i) infections both before and after diagnosis to reflect the clinical reality that many infections are made before being diagnosed in the latent period (13), and (ii) the infection and removal rates being time varying to accommodate changing dynamics of the epidemics.

There are three categories of actions countries can employ as part of the containment strategies: (i) reduce human contact and quarantine the confirmed infected cases to reduce the infectious rate; (ii) increase population virus screening and diagnosis; (iii) better medical treatments that shorten the recovery time from the disease. The three actions' epidemiological effects are well reflected in the expression of the effective reproduction number under the vSEIdR model (11):

$$R_t = \beta_t^E s(t)/\alpha + \beta_t^I s(t)/\gamma_t , \quad (1)$$

where $\beta_t^E$ and $\beta_t^I$ are the infection rates in the pre-diagnosis Exposure state and the Infected state after diagnosis respectively, $\gamma_t$ is the removal rate, $\alpha$ is the diagnostic rate, and s(t) is the proportion of the susceptible population.

The daily counts of infected, dead and recovered cases are obtained from data platforms of Johns Hopkins University (14), World Health Organization (WHO) and Dingxiang Doctor. We did not consider data from China's Hubei province due to the incomplete observation before January 16, 2020. This actually makes the epidemics of the 25 countries more comparable as they are all started with imported cases. The start date for community transmission (DCT) of a country, reported in Table 1, is determined by the first maximum of the estimated infection rate after the WHO local infection date to avoid the early epidemic period caused by imported cases.

The study period is from DCT of each country up to April 20th. The COVID-19 related action date information of the countries is provided in Table S1 in the supplementary data (SD) based on both governmental and credible media sources. When a series of measures are implemented over a time window, we take the average date of the start and the ending dates of the time window as the action time. See Table S1 in SD for specifics. Ten countries have taken actions to counter COVID-19 in less than 13 days from their start dates of local transmission, which are considered as quick action countries, and the other 15 countries as slow action countries.

ORIGINAL UNEDITED MANUSCRIPT

Table 1 reports the estimated $R_t$ (see (11) for the estimation procedure) on the start (t = 0), which can be viewed as the basic reproduction number $R_0$, and the average $R_t$ in Weeks 1-4 and Week 4 since the start date. The $R_t$s measure the underlying reproduction dynamics of the infection beyond the more intuitive statistics, but are dependent with those statistics. Figure S1 in SD presents a scattered plot of the average $R_t$ in Weeks 2-4 and the cases per 100,000 population on April 20th, which shows significant correlation between the two variables. The average $R_0$ among the 25 countries was 5.40 (SE 0.27) with the lower and upper 25% quartiles being 4.57 and 6.26, respectively. One may also use the average $R_t$ in Week 1 to gain information on the force of the epidemic in early dates of the local transmission, which was 4.35 (SE 0.23) among the 25 countries. Our estimates of $R_0$ were higher than most of the $R_0$s from the existing studies on COVID-19, mostly under the SEIR models, for instance 2–3 from (15) and 3.15 (3.04-3.26) in (5) on Wuhan. A reason for our higher $R_0$ is that the vSEIdR model allows infection prior to clinical diagnosis as reflected by the first term of $R_t$ in (1).

Taking quicker containment measures is shown to be effective in reducing the reproduction. Table 1 and Figure 1a show that taking quick control measures reduced the effective reproduction numbers $R_t$ by 0.819 (p-value 0.007) in weeks 2-4 after the start of local transmission between the quick and slow action groups of countries. The reason for comparing only the decline in Weeks 2-4 is to avoid the high volatility in the estimated $R_t$ at the start of the epidemics. The 0.819 decline between the two groups was substantial as the average $R_t$ in Weeks 2-4 were 2.21 among the 25 countries.

China (non-Hubei) and Republic of Korea (South Korea) were the two nations that responded to the COVID-19 emergency the quickest among the 25 countries (see SD), and are found to be the most effective in bringing down the reproduction of COVID-19 in the first four weeks as shown in Table 1 and Figure 1a. COVID-19 epidemic in non-Hubei China had completely lost its force as the average $R_t$ in Week 4 was zero attaining 100% deduction; South Korea's average in Week 4 was 0.2 representing 93% reduction from its $R_0$. The drastic decline in the reproduction of China echoes recent studies on the effectiveness of China's control measures (16, 17). From Table 1, the average $R_t$ in the first four weeks for China and South Korea were sharply less than those of the other 23 countries, with China at 1.03 (95% confidence interval: 0.87-1.2) and Republic of Korea 1.54 (1.43-1.66). In contrast, there were twenty countries whose four week average $R_t$ were larger than 2.0, and ten of them larger than 3.0. Among the twelve countries which had the highest average $R_t$ in the first four weeks of local transmission, nine of them are among the top twelve countries with the most infected cases on June 12, 2020 according to the WHO; the other three countries on the top twelve list had their epidemic started much later than the 25 nations and are not included in our study.

ORIGINAL UNEDITED MANUSCRIPT

Behind South Korea and China's rapid declines in their $R_t$s were two similar but not the same strategies. China's strategy was largely to suppress human contacts by limiting population movement, sealing off cities, enforcing high levels of self-isolation at homes and quarantining confirmed cases in newly built hospitals, which led to rapid decline in the contact rates and then in the infection rates $\beta_t^E$ and $\beta_t^I$ . In addition to limiting population contacts and a quick blockade of Daegu, South Korean conducted more active testing for potential infections in the population with more than half million tests being carried out in the first four weeks (18, 19), which increased the diagnosis rate and hence reduced $R_t$ as implied by (1).

Table 1 also informs that the strongest epidemic force of COVID-19 happened in the European countries with Germany, the UK and Holland having the highest $R_0$ (more than 9.0). Nine out of the fourteen European countries had their $R_0 \geq 5.93$ and were among the ten highest countries. This may be due to the genetic variants of the virus, for instance, the D614G mutation in S protein (20). The five countries with the largest average $R_t$ in Weeks 1-4 were all European countries. The high $R_t$ values were associated with the high death number per 100,000 populations. From WHO, Belgium, the UK, Spain, Italy, Sweden, France, Holland are among the top 9 countries worldwide with the highest death rate as June 12, 2020 (the two others are San Marino and Andorra in southern Europe). All of them had the first four-week average $R_t$ over 2.6, and 6 of them are in the slow action group. The strong epidemic force would require quick and decisive containment actions to counter. Table 1 also shows that just taking quick actions does not guarantee effective control of the epidemic as it depends how the containment measures are enforced. Spain and Brazil were two early action countries. Their implementations were not effective as reflected by their rather high four-week average $R_t$ (Table 1).

Among the three Scandinavian countries Denmark, Norway and Sweden, Norway and Denmark took containment measures in 9 and 11.5 days, respectively, with the effects reflected in the quick declines of $R_t$ of the two countries (Table 1). The Week 4 average $R_t$ were 1.07 and 1.68 for Denmark and Norway, representing 75% and 86% decline from their $R_0$. In contrast, it took Sweden 26 days to put forward an action and its $R_t$ was much larger and slowly declined, with its average $R_t$s in Weeks 2, 3 and 4 hovering near 2. The slow and ineffective actions made Sweden incur larger infection and death rates, which were 478 and 47 per 100,000 populations, respectively, as June 12, 2020 from WHO. In a sharp contrast, Denmark which has more than 5 times population density than Sweden had recorded just over 208 cases and 10 deaths per 100,000 populations, and Norway 159 cases and 5 deaths per 100,000 populations.

One would think that the lead time from China's outbreak of COVID-19 in January to the outbreaks in other countries would provide crucial preparation for the later countries to formulate mitigation strategies and effective measures. To verify if the lead times had been used wisely to curtail the reproduction of COVID-19, we present in Figure 1 b-d weekly reduction rates in Weeks 2-4's average $R_t$ from the

ORIGINAL UNEDITED MANUSCRIPT

average $R_t$ in Week 1 versus the lead times from the DCT of Non-Hubei China (January 23rd) to the DCTs of the other 24 countries. If the lead times were used effectively, one would see a positive correlation between the weekly reduction rates and the lead times. However, Figure 1 b-d do not show significant positive correlation with the correlation coefficients being -0.059, 0.048 and 0.052, respectively, and the p-values all exceeding 0.40. Cares have to be taken when interpreting the above correlation results for causality. However, as causality implies correlation, no correlation means no causality. Hence, as Figure 1b-d all report no significant correlations, this implies that, sadly, most countries have wasted the valuable time to get prepared for the coming of COVID-19 in their countries.

The nations have put forward a set of control measures as summarized in Table 1 supplemented by Table S1 in SD. To evaluate the effectiveness of these measures, we conduct a two sample test for weekly reduction rates in $R_t$ between a High-Level control measure group of eight countries versus the other countries undertaking standard measures. The High-Level control group consists of countries that including four countries (China, Republic of Korea, Italy, Turkey) with the strong lock-down measure together with another four countries (Germany, Malaysia, Holland, Canada) which have implemented at least four measures among a pool of the control measures (Table S1). Although the average weekly reduction rates of $R_t$ were higher in the High-Level control group, no significant differences were detected between the two groups as shown in Table S2 in SD for details.

Our study has two limitations. While the vSEIdR model is more realistic than the SIR and SEIR models, the asymptomatic cases and imported cases are not explicitly accounted for due to lack of data, which may cause bias in the estimation. While allowing infection in the latent stage reduces the bias caused by the asymptomatic cases, deaths and recoveries from asymptomatic cases are still unaccounted for. The bias caused by the imported cases is limited as we choose the DCT to avoid the very early stage of the epidemic largely caused by the imported cases, which is further helped by the fact that cross-country travel has been much dis-encouraged as the first set of counter-measures by countries.

There are several critical lessons one can learn from the 25 countries' COVID-19 experiences. The first one is to take action as early as possible with vigorous enforcement to reduce the contact rates so as to reduce the infection rates and the $R_t$. Acting early vigorously can hugely impact the infection size and thus lessen demands on medical resources down the track, and eventually improve the removal processes for those infected. The second lesson is to maintain a high level of the diagnostic rate to speed up the epidemic progression as favorably shown in Republic of Korea. COVID-19 epidemics are very responsive to early and effective containment measures for the infection rates and the $R_t$ reduction, as well as improved diagnosis. This is largely due to the high infectiousness of COVID-19 virus as reflected by the very high $R_t$ values in the first week of the epidemics among the 25 countries, which

ORIGINAL UNEDITED MANUSCRIPT

leaves room for early containment measures to be effective.

**Acknowledgement** We thank the Section Editor for insightful suggestions that have enriched the content of the work, and three reviewers for beneficial comments and suggestions.

### References

1. Pan A, Liu L and Wang CL et al. Association of Public Health Interventions With the Epidemiology of the COVID-19 Outbreak in Wuhan, China. *JAMA* 2020; **323(19)**: 1915-1923..
2. Zhang JJ, Litvinova M and Wang W. Evolving epidemiology and transmission dynamics of coronavirus disease 2019 outside Hubei province, China: a descriptive and modelling study. *The Lancet Infectious Diseases* 2020; **20(7)**: 793-802.
3. Prem K, Liu Y and Russell T et al. The effect of control strategies to reduce social mixing on outcomes of the COVID-19 epidemic in Wuhan, China: a modelling study. *The Lancet Public Health* 2020; **5(5)**: 261-270.
4. Leung K, Wu JH and Liu D et al. First-wave COVID-19 transmissibility and severity in China outside Hubei after control measures, and second-wave scenario planning: a modelling impact assessment. *The Lancet* 2020; **395(10233)**: 1382–1393.
5. Tian HY, Liu YH and Li YD et al. An investigation of transmission control measures during the first 50 days of the COVID-19 epidemic in China. *Science* 2020; **368(6491)**: 638-642.
6. Chinazzi M, Davis J and Ajelli M et al. The effect of travel restrictions on the spread of the 2019 novel coronavirus (COVID-19) outbreak. *Science* 2020; **368(6489)**: 395-400.
7. Binny R, Hendy S and James A et al. Effect of Alert Level 4 on effective reproduction number: review of international COVID-19 cases. *medRxiv* 2020.
8. Kohanovski I, Obolski U and Ram Y. Inferring the effective start dates of non-pharmaceutical interventions during COVID-19 outbreaks. *medRxiv* 2020.
9. Caicedo-Ochoa Y, Rebellón-Sánchez DE and Peñaloza-Rallón M et al. Effective Reproductive Number estimation for initial stage of COVID-19 pandemic in Latin American Countries. *International Journal of Infectious Diseases* 2020; **95:** 316-318.
10. Linka K, Peirlinck M and Costabal F et al. Outbreak dynamics of COVID-19 in Europe and the effect of travel restrictions. *Computer Methods in Biomechanics and Biomedical Engineering* 2020; **23(11)**: 710-717.
11. Gu J, Yan H and Huang Y et al. Better Strategies for Containing COVID-19 Epidemics — A Study of 25 Countries via an Extended Varying Coefficient SEIR Model. *medRxiv* 2020.
12. Hethcote HW. The mathematics of infectious diseases. *SIAM review* 2000; **42(4)**: 599–653.
13. Guan WJ, Ni ZY and Hu Y et al. Clinical characteristics of coronavirus disease 2019 in China. *New England Journal of Medicine 2020*; **382(18)**: 1708-1720.
14. Dong E, Du H, Gardner L. An interactive web-based dashboard to track COVID-19 in real time. *Lancet Infectious Disease*. **20(5)**: 533-534.
15. Li Q, Guan XH and Wu P et al. Early transmission dynamics in Wuhan, China, of novel coronavirus–infected pneumonia. *New England Journal of Medicine* 2020; **382;** 1199-1207.
16. Maier B and Brockmann D. Effective containment explains subexponential growth in recent confirmed COVID-19 cases in China. *Science* 2020; **368(6492)**: 742-746.
17. Kraemer MUG, Yang CH and Gutierrez B et al. The effect of human mobility and control measures on the COVID-19 epidemic in China. *Science* 2020; **368(6490)**: 493-497.
18. Statista. COVID-19 test case total number South Korea 2020. https:www.statista.com/statistics/1102818/south-korea-covid-19-test-total-number/
19. Normile D. Coronavirus cases have dropped sharply in South Korea. What's the secret to its success? https://www:sciencemag:org/news/2020/03/coronavirus-cases-have-dropped-sharply-southkorea-whats-secret-its-success
20. Korber B, Fischer WM and Gnanakaran S et al. Tracking Changes in SARS-CoV-2 Spike: Evidence that D614G Increases Infectivity of the COVID-19 Virus. *Cell* 2020; **182**: 1-16.

ORIGINAL UNEDITED MANUSCRIPT

Table 1: Weekly averages of the estimated reproduction numbers $R_t$ (W1-W4) of 25 countries over the four weeks from their respective start date of community transmission (DCT), and the average over the first 4 weeks (4W). Time duration shows the four-week interval from DCT. Countries are ranked based on the DCT with the numerical superscripts indicating the types of control measures and the quick action countries are marked in bold. Data of Hubei, Hongkong, Macau and Taiwan are not included in this analysis of China. The 95% confidence intervals for the 4 week averages are reported in the parentheses and those for $R_0$ are available in Table S1 in SM.

| | Country | Time duration | $R_0$ | W1 | W2 | W3 | W4 | 4W-Ave |
|---|---|---|---|---|---|---|---|---|
| 1 | **China**[1,2,3,4,5,6] | 01/23-02/20 | 4.78 | 2.79 | 1.09 | 0.24 | 0.00 | 1.03(0.87-1.2) |
| 2 | Japan[1,2,3] | 02/12-03/11 | 4.83 | 3.17 | 1.79 | 2.07 | 1.63 | 2.17(1.51-2.77) |
| 3 | **Republic of Korea**[2,4,5,6] | 02/17-03/16 | 5.56 | 3.68 | 1.72 | 0.58 | 0.20 | 1.54(1.43-1.66) |
| 4 | **Iran**[2,4,5] | 02/22-03/21 | 8.62 | 5.37 | 2.21 | 1.58 | 1.56 | 2.68(2.37-3.11) |
| 5 | Italy[2,3,6] | 02/23-03/22 | 6.25 | 4.42 | 3.38 | 2.61 | 1.80 | 3.05(2.95-3.18) |
| 6 | France[1,2,3,4] | 02/25-03/24 | 8.57 | 6.13 | 3.92 | 2.95 | 2.49 | 3.87(3.43-4.25) |
| 7 | Germany[2,3,4,5] | 02/25-03/24 | 9.90 | 6.01 | 4.87 | 3.57 | 2.07 | 4.13(3.65-4.71) |
| 8 | UK[2,3,4] | 02/25-03/24 | 9.16 | 5.86 | 4.49 | 3.77 | 3.20 | 4.33(3.82-4.43) |
| 9 | Australia[3,4] | 02/26-03/25 | 4.83 | 3.88 | 3.80 | 3.46 | 2.07 | 3.3(2.84-3.77) |
| 10 | Malaysia[2,3,4,5] | 02/29-03/28 | 4.46 | 3.31 | 3.09 | 1.74 | 1.20 | 2.34(1.21-2.96) |
| 11 | US[1,2,3,5] | 02/29-03/28 | 4.10 | 3.82 | 4.38 | 3.33 | 2.24 | 3.44(3.33-3.51) |
| 12 | Holland[1,2,3,4,5] | 02/29-03/28 | 9.09 | 6.06 | 3.51 | 2.92 | 1.85 | 3.58(3.03-4.11) |
| 13 | **Spain**[1,2,3,4] | 02/29-03/28 | 6.75 | 6.34 | 4.22 | 2.98 | 1.74 | 3.82(3.56-4) |
| 14 | Switzerland[2,3,4] | 03/01-03/29 | 3.64 | 3.46 | 3.08 | 1.89 | 1.20 | 2.41(2.21-2.53) |
| 15 | Sweden[3,4] | 03/01-03/29 | 6.02 | 4.67 | 1.92 | 1.79 | 2.10 | 2.62(2.49-2.72) |
| 16 | **Norway**[2,3,4] | 03/03-03/31 | 4.98 | 4.14 | 1.95 | 1.55 | 1.07 | 2.18(2-2.20) |
| 17 | **Denmark**[1,2,3,4] | 03/03-03/31 | 6.26 | 3.42 | 1.10 | 1.40 | 1.68 | 1.9(1.09-2.39) |
| 18 | Singapore[2,3,4] | 03/04-04/01 | 2.48 | 2.42 | 2.36 | 1.64 | 1.46 | 1.97(1.45-2.36) |
| 19 | Belgium[2,3,5] | 03/04-04/01 | 4.95 | 4.42 | 3.42 | 2.79 | 1.57 | 3.05(2.79-3.3) |
| 20 | **Austria**[2,3,4] | 03/07-04/04 | 3.68 | 3.15 | 2.40 | 1.45 | 0.60 | 1.9(1.21-2.12) |
| 21 | Thailand[1,3,7] | 03/07-04/04 | 4.57 | 4.29 | 2.65 | 1.37 | 0.75 | 2.27(1.84-2.49) |
| 22 | **Canada**[1,2,3,4,5] | 03/07-04/04 | 3.62 | 3.31 | 2.87 | 2.17 | 1.59 | 2.48(2.2-2.61) |
| 23 | **Portugal**[1,2,3] | 03/07-04/04 | 5.93 | 5.09 | 3.21 | 2.10 | 1.20 | 2.9(1.77-4.01) |
| 24 | **Brazil**[1,3,7] | 03/10-04/07 | 5.65 | 4.91 | 2.79 | 2.56 | 1.96 | 3.06(2.9-3.21) |
| 25 | Turkey[6,7] | 03/18-04/15 | 5.54 | 4.59 | 2.60 | 1.98 | 1.66 | 2.83(2.78-2.98) |
| | Ave | | 5.40 | 4.35 | 2.91 | 2.18 | 1.56 | 2.75 |
| | SE | | 0.27 | 0.23 | 0.21 | 0.18 | 0.14 | 0.16 |

[1] State of Emergency; [2] School suspension or closure; [3] Closure of public space or offices;

[4] Restriction on gathering; [5] Asking people to stay at home; [6] Locking down cities;

[7] Imposing curfew; Quick (slow) action countries take action in less (more) than 13 days.

ORIGINAL UNEDITED MANUSCRIPT

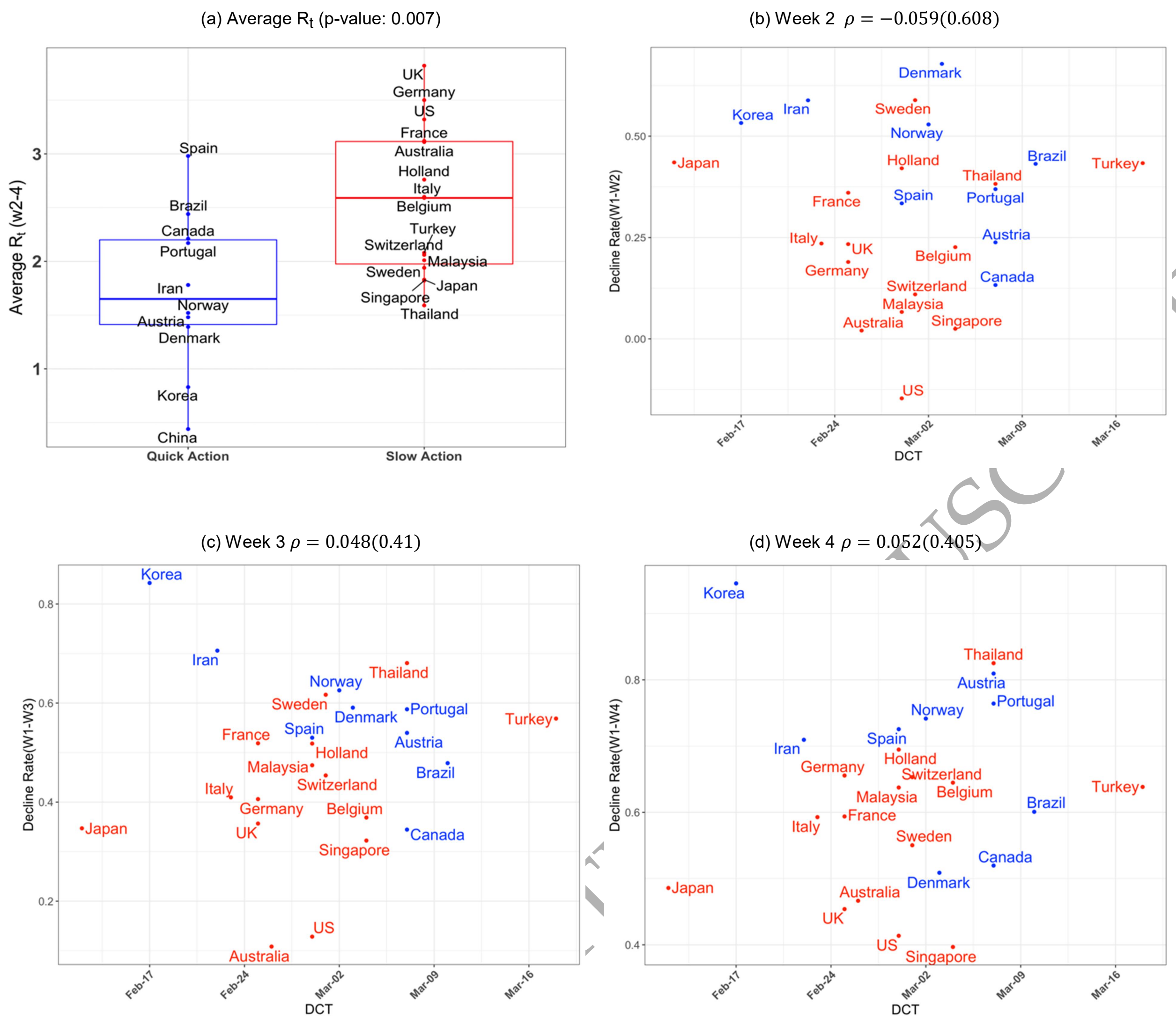

Figure 1: (a) Box plots for the average estimated reproduction numbers $R_t$ in weeks 2-4 since the start date of community transmission (DCT) between the 10 Quick Action countries and the 15 Slow Action countries; (b)–(d) Scatter plots of weekly decline rates in the average effective reproduction number $R_t$ (China excluded) from Week 1 to those of Week 2 (b), Week 3 (c) and Week 4 (d), respectively, versus the lead times (from the DCT of non-Hubei China to the DCT of another country). The header to Panel (a) reports the one-sided two-sample t-statistic (p-values), and those to Panels (b)-(d) report the correlation coefficients (p-values for testing zero correlation) between the declined rates and the lead times among the 24 countries without China. The Quick (Slow) Action countries are marked in blue (red).
