# Comparing containment measures among nations by epidemiological effects of COVID-19

**DOI:** 10.1093/nsr/nwaa243

**Published:** 2020-09-19

**Authors:** Jia Gu, Han Yan, Yaxuan Huang, Yuru Zhu, Haoxuan Sun, Yumou Qiu, Songxi Chen

**Affiliations:** Center for Statistical Science, Peking University, China; Guanghua School of Management, Peking University, China; School of Mathematical Sciences, Sichuan University, China; Yuanpei College, Peking University, China; Center for Statistical Science, Peking University, China; Center for Data Science, Peking University, China; Department of Statistics, Iowa State University, USA; Center for Statistical Science, Peking University, China; Guanghua School of Management, Peking University, China

The coronavirus disease 2019 (COVID-19) has evolved to a global pandemic since its inception in December 2019. Countries have responded to the epidemics with different levels of responses and containment measures. Given the unprecedented pressure on nations’ healthcare systems and the deaths so far, there is an urgent need to evaluate the effects of the containment measures, which would be useful for countries to plan for their responses to counter the first or possibly the second wave of the epidemic. There have been studies for the effects of COVID-19 control measures taken in China on disease transmission and public health interventions for Wuhan's outbreaks [1–4], and Wuhan travel ban on the spread of COVID-19 both inside China and internationally [5,6].

There are cross-country studies on COVID-19 pandemics. The effective reproduction numbers between the early and late phases of COVID-19 outbreaks in 25 international locations (China mainland was not included) were compared with New Zealand's four-tier system in [7]. This approach is based on estimating time-varying growth rate of confirmed cases rather than a dynamic epidemiological model. Estimation of the effective start dates for nonpharmaceutical interventions (NPIs) of 11 European counties and Wuhan, China, was considered in [8], which concluded that the effective start of the NPIs occurred 5 or more days after the official start date of intervention. The effective reproduction numbers of seven Latin American countries were compared with those of Spain and Italy in [9]. This study focused on the first 10 days of the local transmission. The effects of travel restriction on COVID-19 in 27 European countries were analyzed based on a constant coefficient SEIR model and a mobility model in [10].

Our study is focused on 25 countries that had experienced the COVID-19 epidemic earlier in the pandemic such that they have experienced at least 4 weeks of established community infections by 20 April 2020. It is conducted by evaluating and comparing the effective reproduction number *R_t_* curves of the countries and associating them with the timing and the extent of the control measures taken by those countries. The study is based on an extended SEIR model [11] with time-varying coefficients (vSEIdR model). Unlike the conventional SEIR model [12], the vSEIdR model allows (i) infections both before and after diagnosis to reflect the clinical reality that many infections are made before being diagnosed in the latent period [13] and (ii) the infection and removal rates being time varying to accommodate changing dynamics of the epidemics.

There are three categories of actions countries can employ as part of the containment strategies: (i) reduce human contact and quarantine the confirmed infected cases to reduce the infection rate; (ii) increase population virus screening and diagnosis; and (iii) provide better medical treatments that shorten the recovery time from the disease. The three actions’ epidemiological effects are well reflected in the expression of the effective reproduction number under the vSEIdR model [11]:

(1) \begin{equation*} {R_t} = \beta _t^{\rm{E}}s( t )/\alpha + \beta _t^{\rm{I}}s( t )/{\gamma _t}, \end{equation*}

where $\beta _t^{\rm{E}}$ and $\beta _t^{\rm{I}}$ are the infection rates in the pre-diagnosis exposure state and the infected state after diagnosis, respectively, ${\gamma _t}$ is the removal rate,$\ \alpha \ $is the diagnostic rate and *s*(*t*) is the proportion of the susceptible population.

The daily counts of infected, dead and recovered cases are obtained from data platforms of Johns Hopkins University [14], World Health Organization (WHO) and Dingxiang Doctor. We did not consider data from China's Hubei Province due to the incomplete observation before 16 January 2020. This actually makes the epidemics of the 25 countries more comparable as they all started with imported cases. The start date for community transmission (DCT) of a country, reported in Table 1, is determined by the first maximum of the estimated infection rate after the WHO local infection date to avoid the early epidemic period caused by imported cases.

**Table 1.** Weekly averages of the estimated reproduction numbers *R_t_* (W1–W4) of 25 countries over the 4 weeks from their respective DCT, and the average over the first 4 weeks (4W-Ave).

	Country	Time duration	*R*_0_	W1	W2	W3	W4	4W-Ave
1	**China** ^a,b,c,d,e,f^	23 January to 20 February	4.78	2.79	1.09	0.24	0.00	1.03 (0.87–1.2)
2	Japan^a,b,c^	12 February to 11 March	4.83	3.17	1.79	2.07	1.63	2.17 (1.51–2.77)
3	**Republic of Korea** ^b,d,e,f^	17 February to 16 March	5.56	3.68	1.72	0.58	0.20	1.54 (1.43–1.66)
4	**Iran** ^b,d,e^	22 February to 21 March	8.62	5.37	2.21	1.58	1.56	2.68 (2.37–3.11)
5	Italy^b,c,f^	23 February to 22 March	6.25	4.42	3.38	2.61	1.80	3.05 (2.95–3.18)
6	France^a,b,c,d^	25 February to 24 March	8.57	6.13	3.92	2.95	2.49	3.87 (3.43–4.25)
7	Germany^b,c,d,e^	25 February to 24 March	9.90	6.01	4.87	3.57	2.07	4.13 (3.65–4.71)
8	UK^b,c,d^	25 February to 24 March	9.16	5.86	4.49	3.77	3.20	4.33 (3.82–4.43)
9	Australia^c,d^	26 February to 25 March	4.83	3.88	3.80	3.46	2.07	3.3 (2.84–3.77)
10	Malaysia^b,c,d,e^	29 February to 28 March	4.46	3.31	3.09	1.74	1.20	2.34 (1.21–2.96)
11	USA^a,b,c,e^	29 February to 28 March	4.10	3.82	4.38	3.33	2.24	3.44 (3.33–3.51)
12	Netherlands^a,b,c,d,e^	29 February to 28 March	9.09	6.06	3.51	2.92	1.85	3.58 (3.03–4.11)
13	**Spain** ^a,b,c,d^	29 February to 28 March	6.75	6.34	4.22	2.98	1.74	3.82 (3.56–4)
14	Switzerland^b,c,d^	1–29 March	3.64	3.46	3.08	1.89	1.20	2.41 (2.21–2.53)
15	Sweden^c,d^	1–29 March	6.02	4.67	1.92	1.79	2.10	2.62 (2.49–2.72)
16	**Norway** ^b,c,d^	3–31 March	4.98	4.14	1.95	1.55	1.07	2.18 (2–2.20)
17	**Denmark** ^a,b,c,d^	3–31 March	6.26	3.42	1.10	1.40	1.68	1.9 (1.09–2.39)
18	Singapore^b,c,d^	4 March to 1 April	2.48	2.42	2.36	1.64	1.46	1.97 (1.45–2.36)
19	Belgium^b,c,e^	4 March to 1 April	4.95	4.42	3.42	2.79	1.57	3.05 (2.79–3.3)
20	**Austria** ^b,c,d^	7 March to 4 April	3.68	3.15	2.40	1.45	0.60	1.9 (1.21–2.12)
21	Thailand^a,c,g^	7 March to 4 April	4.57	4.29	2.65	1.37	0.75	2.27 (1.84–2.49)
22	**Canada** ^a,b,c,d,e^	7 March to 4 April	3.62	3.31	2.87	2.17	1.59	2.48 (2.2–2.61)
23	**Portugal** ^a,b,c^	7 March to 4 April	5.93	5.09	3.21	2.10	1.20	2.9 (1.77–4.01)
24	**Brazil** ^a,c,g^	10 March to 7 April	5.65	4.91	2.79	2.56	1.96	3.06 (2.9–3.21)
25	Turkey^f,g^	18 March to 15 April	5.54	4.59	2.60	1.98	1.66	2.83 (2.78–2.98)
	Ave		5.40	4.35	2.91	2.18	1.56	2.75
	SE		0.27	0.23	0.21	0.18	0.14	0.16

Time duration shows the 4-week interval from DCT. Countries are ranked based on the DCT with the footnotes indicating the types of control measures and the quick action countries are marked in bold. Data of Hubei, Hong Kong, Macau and Taiwan are not included in this analysis of China. The 95% confidence intervals for the 4-week averages are reported in the parentheses and those for *R*_0_ are available in Table S1 in the Supplementary Data. ^a^State of emergency. ^b^School suspension or closure. ^c^Closure of public space or offices. ^d^Restriction on gathering. ^e^Asking people to stay at home. ^f^Locking down cities. ^g^Imposing curfew; quick (slow) action countries take action in less (more) than 13 days.

The study period is from DCT of each country up to 20 April. The COVID-19-related action date information of the countries is provided in Table S1 in the Supplementary Data based on both governmental and credible media sources. When a series of measures are implemented over a time window, we take the average date of the start and the ending dates of the time window as the action time. See Table S1 in the Supplementary Data for specifics. Ten countries have taken actions to counter COVID-19 in <13 days from their start dates of local transmission, which are considered as quick action countries, and the other 15 countries are considered as slow action countries.

Table 1 reports the estimated *R_t_* (see [11] for the estimation procedure) on the start (*t* = 0), which can be viewed as the basic reproduction number *R*_0_, and the average *R_t_* in Weeks 1–4 and Week 4 since the start date. The *R_t_* values measure the underlying reproduction dynamics of the infection beyond the more intuitive statistics, but are dependent on those statistics. Figure S1 in the Supplementary Data presents a scatter plot of the average *R_t_* in Weeks 2–4 and the cases per 100 000 population on 20 April, which shows significant correlation between the two variables. The average *R*_0_ among the 25 countries was 5.40 (standard error [SE] 0.27) with the lower and upper 25% quartiles being 4.57 and 6.26, respectively. One may also use the average *R_t_* in Week 1 to gain information on the force of the epidemic in early dates of the local transmission, which was 4.35 (SE 0.23) among the 25 countries. Our estimates of *R*_0_ were higher than most of the *R*_0_ values from the existing studies on COVID-19, mostly under the SEIR models, for instance, 2–3 from [15] and 3.15 (3.04–3.26) in [5] on Wuhan. A reason for our higher *R*_0_ is that the vSEIdR model allows infection prior to clinical diagnosis as reflected by the first term of *R_t_* in [1].

Taking quicker containment measures is shown to be effective in reducing the reproduction. Table 1 and Fig. 1a show that taking quick control measures reduced the effective reproduction numbers *R_t_* by 0.819 (*P*-value 0.007) in Weeks 2–4 after the start of local transmission between the quick and slow action groups of countries. The reason for comparing only the decline in Weeks 2–4 is to avoid the high volatility in the estimated *R_t_* at the start of the epidemics. The decline of 0.819 between the two groups was substantial as the average *R_t_* in Weeks 2–4 was 2.21 among the 25 countries.

China (non-Hubei) and Republic of Korea (South Korea) were the two nations that responded to the COVID-19 emergency the quickest among the 25 countries (see the Supplementary Data), and are found to be the most effective in bringing down the reproduction of COVID-19 in the first 4 weeks as shown in Table 1 and Fig. 1a. COVID-19 epidemic in non-Hubei China had completely lost its force as the average *R_t_* in Week 4 was zero attaining 100% deduction; South Korea's average in Week 4 was 0.2, representing 93% reduction from its *R*_0_. The drastic decline in the reproduction of China echoes recent studies on the effectiveness of China's control measures [16,17]. From Table 1, the average *R_t_* values in the first 4 weeks for China and South Korea were sharply less than those of the other 23 countries, with China at 1.03 (95% confidence interval: 0.87–1.2) and Republic of Korea at 1.54 (1.43–1.66). In contrast, there were 20 countries whose 4-week average *R_t_* values were >2.0, and 10 of them were >3.0. Among the 12 countries that had the highest average *R_t_* in the first 4 weeks of local transmission, nine of them were among the top 12 countries with the most infected cases on 12 June 2020 according to the WHO; the other 3 countries on the top 12 list had their epidemic started much later than the 25 nations and are not included in our study.

**Figure 1.**
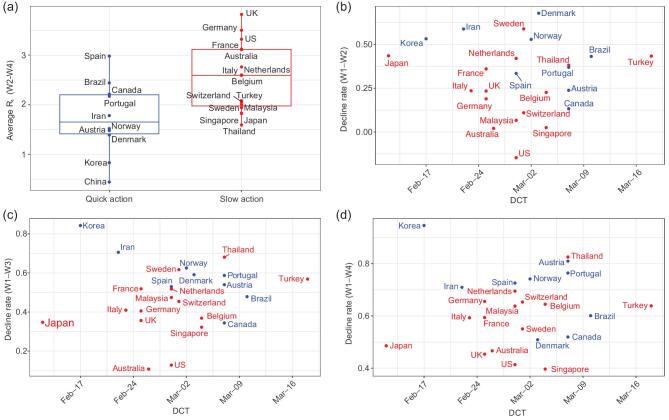
(a) Box plots for the average estimated reproduction numbers *R_t_* in Weeks 2–4 since the DCT between the 10 quick action countries and the 15 slow action countries. Scatter plots of weekly decline rates in the average effective reproduction number *R_t_* (China excluded) from Week 1 to those of Week 2 (b), Week 3 (c) and Week 4 (d), respectively, versus the lead times (from the DCT of non-Hubei China to the DCT of another country). The header to panel (a) reports the one-sided two-sample *t*-statistic (*P*-values), and those to panels (b)–(d) report the correlation coefficients (*P*-values for testing zero correlation) between the declined rates and the lead times among the 24 countries without China. The quick (slow) action countries are marked in blue (red).

Behind South Korea and China's rapid declines in their *R_t_* values were two similar but not the same strategies. China's strategy was largely to suppress human contacts by limiting population movement, sealing off cities, enforcing high levels of self-isolation at homes and quarantining confirmed cases in newly built hospitals, which led to rapid decline in the contact rates and then in the infection rates $\beta _t^{\rm{E}}$ and $\beta _t^{\rm{I}}$. In addition to limiting population contacts and a quick blockade of Daegu, South Korea conducted more active testing for potential infections in the population with more than half million tests being carried out in the first 4 weeks [18,19], which increased the diagnosis rate and hence reduced *R_t_* as implied by [1].

Table 1 also informs that the strongest epidemic force of COVID-19 happened in the European countries with Germany, the UK and Netherlands having the highest *R*_0_ (>9.0). Nine out of the 14 European countries had their *R*_0_ ≥ 5.93 and were among the 10 highest countries. This may be due to the genetic variants of the virus, for instance, the D614G mutation in S protein [20]. The five countries with the largest average *R_t_* in Weeks 1–4 were all European countries. The high *R_t_* values were associated with the high death number per 100 000 populations. From WHO, Belgium, the UK, Spain, Italy, Sweden, France and Netherlands were among the top nine countries worldwide with the highest death rate as of 12 June 2020 (the two others were San Marino and Andorra in southern Europe). All of them had the first 4-week average *R_t_* over 2.6, and six of them were in the slow action group. The strong epidemic force would require quick and decisive containment actions to counter. Table 1 also shows that just taking quick actions does not guarantee effective control of the epidemic as it depends on how the containment measures are enforced. Spain and Brazil were two early action countries. Their implementations were not effective as reflected by their rather high 4-week average *R_t_* (Table 1).

Among the three Scandinavian countries Denmark, Norway and Sweden, Norway and Denmark took containment measures in 9 and 11.5 days, respectively, with the effects reflected in the quick declines of *R_t_* of the two countries (Table 1). The Week 4 average *R_t_* values were 1.07 and 1.68 for Denmark and Norway, representing 75% and 86% decline from their *R*_0_. In contrast, it took Sweden 26 days to put forward an action and its *R_t_* was much larger and slowly declined, with its average *R_t_* values in Weeks 2, 3 and 4 hovering near 2. The slow and ineffective actions made Sweden incur larger infection and death rates, which were 478 and 47 per 100 000 populations, respectively, as of 12 June 2020 from WHO. In a sharp contrast, Denmark that has >5 times population density than Sweden had recorded just over 208 cases and 10 deaths per 100 000 populations, and Norway 159 cases and 5 deaths per 100 000 populations.

One would think that the lead time from China's outbreak of COVID-19 in January to the outbreaks in other countries would provide crucial preparation for the later countries to formulate mitigation strategies and effective measures. To verify whether the lead times had been used wisely to curtail the reproduction of COVID-19, we present in Fig. 1b–d weekly reduction rates in Weeks 2–4’s average *R_t_* from the average *R_t_* in Week 1 versus the lead times from the DCT of non-Hubei China (23rd January) to the DCTs of the other 24 countries. If the lead times were used effectively, one would see a positive correlation between the weekly reduction rates and the lead times. However, Figure 1b–d does not show significant positive correlation with the correlation coefficients being −0.059, 0.048 and 0.052, respectively, and the *P*-values all exceeding 0.40. Care has to be taken when interpreting the above correlation results for causality. However, as causality implies correlation, no correlation means no causality. Hence, as Fig. 1b–d reports no significant correlations, this implies that, sadly, most countries have wasted the valuable time to get prepared for the coming of COVID-19 in their countries.

The nations have put forward a set of control measures as summarized in Table 1 supplemented by Table S1 in the Supplementary Data. To evaluate the effectiveness of these measures, we conduct a two-sample test for weekly reduction rates in *R_t_* between a high-level control measure group of eight countries versus the other countries undertaking standard measures. The high-level control group consists of four countries (China, Republic of Korea, Italy, Turkey) with the strong lockdown measure together with another four countries (Germany, Malaysia, Netherlands, Canada) that have implemented at least four measures among a pool of the control measures (Table S1 in the Supplementary Data). Although the average weekly reduction rates of *R_t_* were higher in the high-level control group, no significant differences were detected between the two groups as shown in Table S2 in the Supplementary Data for details.

Our study has two limitations. While the vSEIdR model is more realistic than the SIR and SEIR models, the asymptomatic cases and imported cases are not explicitly accounted for due to lack of data, which may cause bias in the estimation. While allowing infection in the latent stage reduces the bias caused by the asymptomatic cases, deaths and recoveries from asymptomatic cases are still unaccounted for. The bias caused by the imported cases is limited as we choose the DCT to avoid the very early stage of the epidemic largely caused by the imported cases, which is further helped by the fact that cross-country travel has been much discouraged as the first set of countermeasures by countries.

There are several critical lessons one can learn from the 25 countries’ COVID-19 experiences. The first one is to take action as early as possible with vigorous enforcement to reduce the contact rates so as to reduce the infection rates and the *R_t_*. Acting early vigorously can hugely impact the infection size and thus lessen demands on medical resources down the track, and eventually improve the removal processes for those infected. The second lesson is to maintain a high level of the diagnostic rate to speed up the epidemic progression as favorably shown in Republic of Korea. COVID-19 epidemics are very responsive to early and effective containment measures for the infection rates and the *R_t_* reduction, as well as improved diagnosis. This is largely due to the high infectiousness of COVID-19 virus as reflected by the very high *R_t_* values in the first week of the epidemics among the 25 countries, which leaves room for early containment measures to be effective.

## Supplementary Material

nwaa243_Supplemental_File

## References

[1] Pan A, Liu L, Wang CL et al. JAMA 2020; 323: 1915–23. 10.1001/jama.2020.6130 32275295 PMC7149375

[2] Zhang JJ, Litvinova M, Wang W. Lancet Infect Dis 2020; 20: 793–802. 10.1016/S1473-3099(20)30230-9 32247326 PMC7269887

[3] Prem K, Liu Y, Russell T et al. Lancet Public Health 2020; 5: 261–70. 10.1016/S2468-2667(20)30073-6

[4] Leung K, Wu JH, Liu D et al. Lancet North Am Ed 2020; 395: 1382–93. 10.1016/S0140-6736(20)30746-7 PMC7195331 32277878

[5] Tian HY, Liu YH, Li YD et al. Science 2020; 368: 638–42. 10.1126/science.abb6105 32234804 PMC7164389

[6] Chinazzi M, Davis J, Ajelli M et al. Science 2020; 368: 395–400. 10.1126/science.aba9757 32144116 PMC7164386

[7] Binny R, Hendy S, James A et al. Effect of alert level 4 on effective reproduction number: review of international COVID-19 cases. medRxiv, doi: 10.1101/2020.04.30.20086934, 2020.

[8] Kohanovski I, Obolski U, Ram Y. Inferring the effective start dates of non-pharmaceutical interventions during COVID-19 outbreaks. medRxiv, doi: 10.1101/2020.05.24.20092817, 2020. 10.1016/j.ijid.2021.12.364 PMC8720386 34986406

[9] Caicedo-Ochoa Y, Rebellón-Sánchez DE, Peñaloza-Rallón M et al. Int J Infect Dis 2020; 95: 316–8. 10.1016/j.ijid.2020.04.069 32360941 PMC7192078

[10] Linka K, Peirlinck M, Costabal F et al. Comput Methods Biomech Biomed Eng 2020; 23: 710–7. 10.1080/10255842.2020.1759560 PMC7429245 32367739

[11] Gu J, Yan H, Huang Y et al. Better strategies for containing COVID-19 epidemics: a study of 25 countries via an extended varying coefficient SEIR model. medRxiv, doi: 10.1101/2020.04.27.20081232, 2020.

[12] Hethcote HW. SIAM Rev 2000; 42: 599–653. 10.1137/S0036144500371907

[13] Guan WJ, Ni ZY, Hu Y et al. N Engl J Med 2020; 382: 1708–20. 10.1056/NEJMoa2002032 32109013 PMC7092819

[14] Dong E, Du H, Gardner L. Lancet Infect Dis 2020; 20: 533–4. 10.1016/S1473-3099(20)30120-1 32087114 PMC7159018

[15] Li Q, Guan XH, Wu P et al. N Engl J Med 2020; 382; 1199–207. 10.1056/NEJMoa2001316 31995857 PMC7121484

[16] Maier B, Brockmann D. Science 2020; 368: 742–6. 10.1126/science.abb4557 32269067 PMC7164388

[17] Kraemer MUG, Yang CH, Gutierrez B et al. Science 2020; 368: 493–7. 10.1126/science.abb4218 32213647 PMC7146642

[18] Statista . COVID-19 test case total number: South Korea, 2020. https:www.statista.com/statistics/1102818/south-korea-covid-19-test-total-number/ (1 September 2020, date last accessed).

[19] Normile D. Science 2020: doi: 10.1126/science.abb7566 (1 September 2020, date last accessed).

[20] Korber B, Fischer WM, Gnanakaran S et al. Cell 2020; 182: 1–16. 10.1016/j.cell.2020.06.043 32649872

