# Supplementary material for: Comparing containment measures among nations by epidemiological effects of COVID-19
Source: Natl Sci Rev. 2020 Sep 19;7(12):1847–51. doi: 10.1093/nsr/nwaa243 (PMC7543445; doi:10.1093/nsr/nwaa243)
Supplement: nwaa243_Supplemental_File [file nwaa243_supplemental_file.pdf]

Supplementary Data to ”Comparing the Containment Measures among Nations by the Epidemiological Effects of COVID-19”

Jia Gu,[1] Han Yan,[2] Yaxuan Huang,[3] Yuru Zhu,[1] Haoxuan Sun,[4]
Yumou Qiu,[5*] Song Xi Chen[61*]

[1]Center for Statistical Science, Peking University
[2]School of Mathematical Sciences, Sichuan University
[3]Yuanpei College, Peking University
[4]Center for Data Science, Peking University
[5]Department of Statistics, Iowa State University
[6]Guanghua School of Management, Peking University

*To whom correspondence should be addressed S. X. Chen at csx@gsm.pku.edu.cn and
Y. Qiu at yumouqiu@iastate.edu

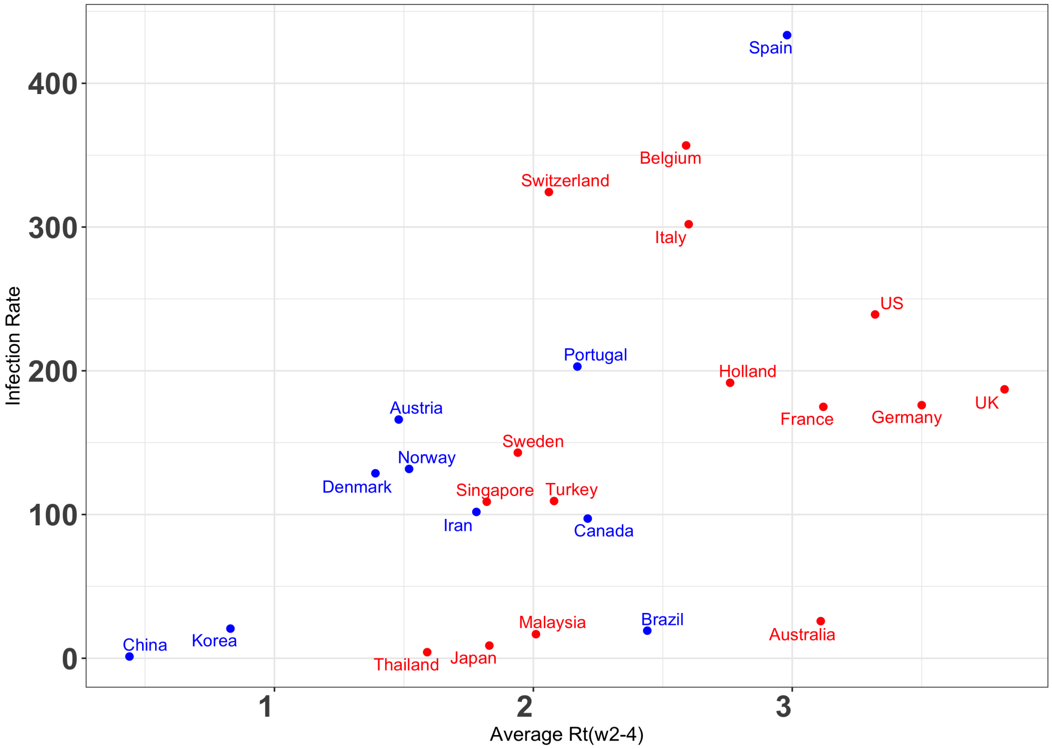

Figure S1: Scatter plot of positive cases per 100,000 population (till April 20th, 2020) versus the estimated average reproduction number R_t in Weeks 2-4 since the start date of community transmission among the 25 countries with the quick action countries in blue and slow action countries in red. The correlation was 0.48 with p-value 0.008 for the one-sided alternative against the null hypothesis of zero correlation.

Table S1: Key dates for taking COVID-19 control measures for the 25 countries and R_0 (the 95% confidence interval). DCT: start date of community transmission. When a country's control measures are phasing in over a time window, we provide the first and the last dates; otherwise, the first date is the date of action.

	Country	DCT	R_0(95% CI)	Response time	Control measures	
					First date	Last date
1	Australia[3,4]	02-26	4.83(3.75-8.54)	30.5	03-25	03-30
2	Austria[2,3,4]	03-07	3.68(1.58-4.85)	6	03-10	03-16
3	Belgium[2,3,5]	03-04	4.95(2.55-6.68)	14	03-18	
4	Brazil[1,3,7]	03-10	5.65(4.32-6.91)	10.5	03-18	03-28
5	Canada[1,2,3,4,5]	03-07	3.62(2.10-4.60)	12.5	03-17	03-22
6	China[1,2,3,4,5,6]	01-23	4.78(3.15-6.57)	2	01-25	
7	Denmark[1,2,3,4]	03-03	6.26(4.98-7.94)	11.5	03-11	03-18
8	France[1,2,3,4]	02-25	7.03(3.46-13.92)	20	03-13	03-19
9	Germany[2,3,4,5]	02-25	6.43(3.15-11.52)	20	03-10	03-22
10	Holland[1,2,3,4,5]	02-29	6.74(2.97-28.57)	17	03-12	03-22
11	Iran[2,4,5]	02-22	8.62(2.92-20.24)	10	02-28	03-07
12	Italy[2,3,6]	02-23	6.25(4.69-8.52)	19.5	03-04	03-23
13	Japan[1,2,3]	02-12	4.83(2.74-9.62)	19	03-02	
14	Korea[2,4,5,6]	02-17	5.56(3.93-7.23)	8	02-25	
15	Malaysia[2,3,4,5]	02-29	4.46(4.05-8.52)	18	03-18	
16	Norway[2,3,4]	03-03	5.21(5.03-5.38)	9	03-12	
17	Portugal[1,2,3]	03-07	5.93(4.99-10.30)	9.5	03-15	03-18
18	Singapore[2,3,4]	03-04	2.48(1.45-3.81)	30	04-03	
19	Spain[1,2,3,4]	02-29	6.75(4.52-8.73)	11.5	03-09	03-14
20	Sweden[3,4]	03-01	6.02(5.23-6.65)	26	03-27	
21	Switzerland[2,3,4]	03-01	3.64(2.30-4.64)	15.5	03-13	03-20
22	Thailand[1,3,7]	03-07	4.57(1.44-6.40)	23	03-26	04-07
23	Turkey[6,7]	03-18	5.54(4.93-6.02)	19.5	04-03	04-10
24	UK[2,3,4]	02-25	7.03(2.06-14.29)	24.5	03-18	03-23
25	US[1,2,3,5]	02-29	4.10(3.15-4.76)	13	03-13	
	Ave(SE)		5.40(0.27)	16(1.46)		

[1] State of Emergency; [2] School suspension or closure; [3] Closure of public space or offices;
[4] Restriction on gathering; [5] Asking people to stay at home; [6] Locking down cities; [7] Imposition of a curfew;

Comparison of Control Measures

Six control measures have been taken into consideration by the 25 countries: Locking down cities, school suspension or closure, closure of public space or offices, restriction on gathering, asking people to stay at home, imposition of a curfew. We included the four countries which have implemented the lock-down policy (China, Korea, Italy, Turkey) together with another four countries (Germany, Malaysia, Holland, Canada) which have implemented at least four actions among a pool of the control measures as the High-Level control group. We did not consider the national emergency as a measure as it needs specific policy measures to back up. The other countries are classified as the Usual-Level control group. Table S2 reports the differences in the weekly R_t reduction rates from Week 1's average R_t to Weeks 2–4 between the two groups. Although the neat reduction rates were positive, they were not significantly away from zero statistically at 5%. We have also experimented finer division of the usual group to medium and low action groups. The results were also not significant.

	High-Usual
Week1-Week2	0.010(0.46)
Week1-Week3	0.092(0.15)
Week1-Week4	0.102(0.08)

Table S2: Differences of mean decline rates of effective reproduction number R_t between the High-Level control countries and the Usual-Level control countries. P-values of one-sided t-test are reported in the parentheses with alternative hypothesis being true difference is greater than 0. High-Level control: China, Korea, Italy, Turkey, Germany, Malaysia, Holland, Canada; Usual-Level control: the other 17 countries.
